# ENED-GEM: A Conceptual Framework Model for Psychological Enjoyment Factors and Learning Mechanisms in Educational Games about the Environment

**DOI:** 10.3389/fpsyg.2017.01085

**Published:** 2017-06-28

**Authors:** Kristoffer S. Fjællingsdal, Christian A. Klöckner

**Affiliations:** Department of Psychology, Norwegian University of Science and TechnologyTrondheim, Norway

**Keywords:** educational games, environmental games, motivation, immersion, flow, semantic memory, episodic memory, perceived behavioral control

## Abstract

Based on a thorough review of psychological literature, this article seeks to develop a model of game enjoyment and environmental learning (ENvironmental EDucational Game Enjoyment Model, ENED-GEM) and delineate psychological processes that might facilitate learning and inspire behavioral change from educational games about the environment. A critically acclaimed digital educational game about environmental issues (Fate of the World by Red Redemption/Soothsayer Games) was used as a case study. Two hundred forty-nine reviews of the game from the popular gaming and reviewing platform known as *Steam* were analyzed by means of a thematic content analysis in order to identify key player enjoyment factors believed to be relevant to the process of learning from games, as well as to gain an understanding of positive and negative impressions about the game’s general content. The end results of the thematic analysis were measured up to the suggested ENED-GEM framework. Initial results generally support the main elements of the ENED-GEM, and future research into the importance of these individual core factors is outlined.

As educational games grow more sophisticated and subject-specific, new models for understanding their influence on human learning are required. In environmental communication, the use of games is considered an innovative and highly specialized method of reaching out to a new and growing media audience about the various global issues we might be facing. In the case of videogames in particular, an estimated 65% of U.S. households alone are home to at least one person who plays regularly for 3 h or more per week (ESA, 2017). Older numbers from a study encompassing eight major European nations suggest that about 25% of adults have played a videogame in the past 6 months, and that approximately 95.2 million adult gamers were divided across the 18 countries covered in the survey (ISFE, 2010). Both of these reports suggest that there is a relatively even distribution of gamers in regards to age and gender (ISFE, 2010; ESA, 2016). Board games, on the other hand, are commonly played on mobile phones and have thus become considerably more digitized, although individuals who regularly play videogames tend to play board games less often (ESA, 2016). However, websites such as Boardgamegeek.com have been established in order to let people review, trade, discuss, and chat about tabletop gaming.

An educational game is commonly defined as any type of game that wants to do more than just entertain the player, normally by increasing certain fields of knowledge or teaching new skills through gameplay (Griffiths, 2002; Barab et al., 2010). Considering educational games as *microworlds* might help to understand how games might contribute to these forms of learning. A microworld is commonly described as a small domain of interest where the degree of immersion into the subject is particularly high (Rieber, 1996). When subjected to such microworlds, learners are encouraged to obtain information and skills on their own volition, in what is called *self-regulated learning* (Zimmermann, 1990). Educational games fit the definition of microworlds in that they usually portray a small domain in which the learner is immersed and encouraged to achieve some form of learning outcome, normally in the form of increased knowledge about a topic or perhaps even behavioral change.

In research literature, educational games are also known under a wide variety of different names, such as serious games or transformational games. These terminologies are often used interchangeably, even leading some researchers to suggest the development of an all-fitting descriptive category (Schmidt et al., 2015).

Educational games need to be considered as conglomerates of different genres and game types (Riemer and Schrader, 2015). Due to the large variety of educational games available on the market, it is likely that there are several psychological factors in play that vary across game types and facilitate learning in the players. In order to understand the potential impact of these factors, it is important to consider the interaction between the gaming audience as well as the interactive, motivational and entertaining aspects a game usually consists of. This article attempts to generate an understanding of the psychological factors that facilitate learning, enjoyment and their interaction in *environmental* educational games, and present these in a conceptual framework for future studies, which we like to refer to as the *ENED-GEM* (ENvironmental EDucational Game Enjoyment Model).

In this article we will present existing research on how games are utilized in educational contexts. Then we shall attempt to put forth the initial suggestions for how the ENED-GEM framework is structured, as well as to highlight central psychological processes that occur before and during gameplay and facilitate learning. Then, in order to provide preliminary evidence for the suggested ENED-GEM framework, a thematic analysis was conducted on reviews of a modern environmental game to identify some of the proposed elements of the model. Lastly, limitations of the study as well as potential future research guidelines are highlighted.

## Game-Based Learning and the Environment

Games and simulations have long been successfully used as educational tools within a wide variety of fields, ranging from geography (Tüzün et al., 2009) to medical education (Gutiérrez et al., 2007) and industrial engineering (Braghirolli et al., 2016). Games such as the ones studied in these papers primarily seek to increase the player’s knowledge, or to positively affect the player’s level of intrinsic motivation to learn. Meta-analyses on the effects of educational gaming tend to reveal mixed to positive findings (e.g., Vogel et al., 2006; Ke, 2009; DeSmet et al., 2014), suggesting that implementing educational games requires a careful consideration of contextual variables. Additionally, very few game-based learning tools are focused on the environment (Klöckner, 2015, p. 200), although the number of sophisticated environmental games has steadily increased since the earliest known publication on the subject in 1983 (Reckien and Eisenack, 2013).

While games exist in many formats, this paper primarily considers digital games and board games when accounting for educational value. This is due to the large body of scientific literature proving the efficiency of these types of games in other learning contexts, as well as the fact that digital games and board games often share significant similarities in design and layout. Educational videogames tend to be immersive learning experiences that attract wide audiences, and allow players to set goals and interact with the game environment experimentally without having to worry about failure (Griffiths, 2002). Studies focusing on environmentally oriented videogames also suggest that games can be attention-grabbing as well as tools for initiating discussions about complex environmental topics. One example includes *LandYOUs*, a game designed to teach the players about sustainable land management and the utilization of limited resources (Schulze et al., 2015). In regards to board games, where research is slightly more limited than in the case of digital games, positive learning outcomes from playing them have been observed across a wide range of subjects (e.g., Ogershok and Cottrell, 2004; Amaro et al., 2006; Eisenack, 2012). Within environmental research, board games such as *CO^2^* and the Oil Expansion pack of *Settlers of Catan* are perhaps the most well-known educational games, featuring such topics as pollution, biofuel and the use of oil, just to name a few.

## Player Enjoyment, Motivations and Game-Based Learning

Player enjoyment is a highly complex and multifaceted psychological construct known to be significantly related to a pleasurable gameplay experience as well as positive learning outcomes. Examples include increasing personal skills such as visual short-term memory (e.g., Boot et al., 2008), and general knowledge structures (Gee, 2003; Fu et al., 2009) as well as contributing to a higher degree of mental well-being (Schell, 2008; Johnson et al., 2013, p. 442). Player enjoyment generally stems from perceiving a game as ‘fun,’ which in turn could be defined as the essence of play in general (Huizinga, 1938–2014, p. 3). An environmental game that is considered fun or enjoyable to play would also likely provide a strong foundation for intrinsic motivation to keep playing and learning from it (Bisson and Luckner, 1996). On the other hand, if a game is not considered fun or enjoyable, nobody wants to play it (Sweetser and Wyeth, 2005). Within the field of learning, perceiving a topic as boring will result in a decline in learning outcomes (De Baker et al., 2010). To conclude, the quality of educational games is directly relatable to the quality of the learning that takes place (McCallum, 2012) as well as the player’s voluntary interaction with the game itself. To elaborate, environmental games need to be *perceived* as “good” by the player in order for intrinsically motivated play and subsequent learning outcomes to occur. Such motivational factors to play are well-known in the commercial game industry. Therefore, a good educational game should aim to capture the player’s attention while simultaneously applying the same motivational elements that commercially successful games tend to do.

While the list of such elements is extensive, examples according to Sweetser and Wyeth (2005) include a sufficient level of challenge, having players feel a degree of control over the game they play, appropriate feedback on how close the player is to achieving their goal and even the ability to cooperate and interact with other players. Together, these elements should lead to a higher degree of player enjoyment, which becomes paramount when applied to the subgenre of *serious games*. The term “serious games” is generally used synonymously with educational games, and refers to games that seek to increase knowledge and alter behavior (Connolly et al., 2012) where in-game content can transfer to real-world experience through repeated play (Bogost, 2010, p. 236). If a game does not engage the player from the very start, it is likely that such repeated play will not occur (Sweetser and Wyeth, 2005). Due to the interplay between enjoyment, immersion and good learning, it is likely that the game needs to be enjoyable or pleasurable to the person playing it in order for the learning outcomes to be high.

Player enjoyment has been the focus of several psychological frameworks attempting to understand its importance in regards to individuals’ motivations to play. Examples include the GameFlow model (Sweetser and Wyeth, 2005) and its derivative EGameFlow scale (Fu et al., 2009). The GameFlow model states that player enjoyment stems from eight primary categories of gameplay, ranging from more visual in-game elements such as attention-grabbing and immersive stimuli, to smooth and operable game mechanics. The EGameFlow tool added knowledge improvement to this model, and is utilized for the evaluation of e-learning games where increasing the player’s semantic memory in some way is the intended outcome. This framework is highly comprehensive and serves as a useful tool in game design, and the ENED-GEM is an attempt to further conceptualize the potential path to learning through gameplay, with a special emphasis on how environmental games can provide increased levels of knowledge and perceived behavioral control over environmental topics.

While the body of literature on player enjoyment is growing, there is still a lack of player enjoyment models dedicated to educational games about the environment. Educational games seek to increase the player’s knowledge, skills, involvement or interest in a given topic, usually through presenting this topic in an attractive and highly immersive context (Barab et al., 2010). Environmental games almost certainly contain some of the traditionally enjoyable elements found in other types of educational games. However, they should also aim to have a measureable positive effect on the players’ motivation to perform some kind of pro-environmental behavior in order to be considered effective. Furthermore, the call for research into different types of educational games, environmental ones included, has been made (Riemer and Schrader, 2015). This article seeks to present the ENED-GEM as an example of such an attempt and clarify its potential role in the design and development of entertaining and educational environmental games, as well as to provide insight into how certain psychological constructs and processes important to behavioral change can be facilitated and strengthened by playing.

## Focused Environmental Themes in Games and Ideal Level of Information

According to the Tbilisi Declaration (UNESCO, 1978), responsible environmental behavior needs to be outlined, detailed, and explained in a fashion understandable to the major public. While games can be capable of increasing an individual’s level of knowledge about an environmental topic according to these guidelines, one of the biggest challenges for game designers in implementing environmental themes in educational games is the high complexity of environmental issues (Fennewald and Kievit-Kylar, 2012). If a game presents too much information at once, which would likely be the case if several environmental issues are outlined and intended to be overcome simultaneously, the player would likely suffer from cognitive overload due to how complex environmental issues are. This form of information overload is generally considered to be one of the most detrimental factors in computer-based learning (Chen et al., 2011). Furthermore, providing environmental information alone generally does not lead to behavioral change unless the information provided is highly tailored to the recipients (Abrahamse et al., 2005) or is highly specific in nature (Klöckner, 2015, p. 165). However, it should be noted that this is likely just the case of educational games focused toward increasing some aspect of knowledge. Games can also enable learners to acquire new skills, teach complex problem-solving and even experience emotional journeys where they can identify with or even adopt traits from the characters they encounter in the virtual world (Klimmt et al., 2009).

A promising strategy to avoid the issue of information overload in particular is to design games dedicated to singular faceted environmental issues rather than to focus on the full environmental picture, such as focusing on *biodiversity problems* rather than the general moniker of *environmental problems* (e.g., Sandbrook et al., 2015). Designing a thematically focused game would allow the player to allocate cognitive resources toward solving one manageable problem rather than dedicate their attention toward too many variables at once. In gameplay, the tendency for games to demand that the player directs their attention toward a large quantity of in-game variables at once is called *micromanagement*. A high degree of micromanagement might detract from the player’s ability to learn from environmentally oriented games in favor of having to keep up with the game’s progression or memorize unnecessary details. A lower degree of micromanagement in educational games allows the player to focus more on the environmental issue being presented.

Understanding the issue as well as the tools required to overcome it could ideally result in a higher degree of *perceived behavioral control* (PBC), or the degree to which a behavior is perceived as easy or difficult to perform (Ajzen, 2002). PBC is commonly considered a central determinant for behavioral change. A game designed in this manner should also provide a higher degree of tailored information and feedback according to the individual’s chosen play style, which in other contexts has been shown to have a positive impact on pro-environmental behavior (e.g., Abrahamse et al., 2007). In so doing, it should aim to present the topic in a novel way, ideally by appearing as personally relevant to the player. Novel strategies for communication are known to increase interest in the topic through encouraging the individual to approach the phenomenon in question from a different angle than what is common or familiar. When such novel forms of communication strategies succeed, there is reason to believe that the individual will be motivated to seek out more knowledge about the topic willingly, as well as to expand upon knowledge they already possess (Ainley et al., 2002). Additionally, perceiving topical information as personally relevant is known to have a significant impact upon peoples’ attitudes toward specific sustainability issues (Kang et al., 2013).

## The ENED-GEM

**FIGURE 1:**
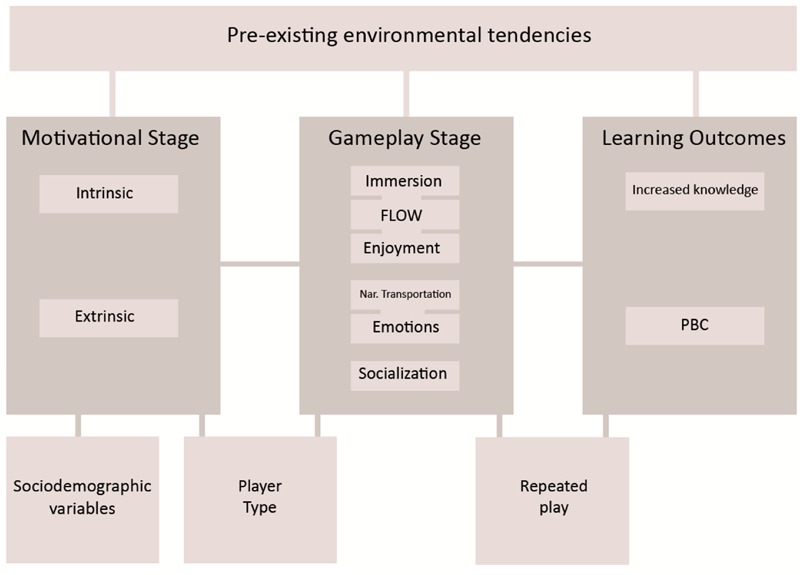
The ENED-GEM framework.

As Riemer and Schrader (2015) point out, new models for understanding and designing educational games are required. One way to answer this call is to design more subject-oriented conceptual models for educational games, where variables related to the topic at hand are put into focus. In the case of educational games about environmental issues and subjects, there is currently no such model available in existing research. Also, considering the unique complexity of understanding environmental topics, as explained by Klöckner (2015) as well as Fennewald and Kievit-Kylar (2012), it would make sense to develop a model for this exact purpose. Therefore, we suggest the ENED-GEM as a potential explanatory framework for the design and implementation of environmental games. In order to establish a prototypical framework, existing literature about educational and environmental games were retrieved, and central recurring factors in said literature were implemented in the ENED-GEM. Insight from fields such as media psychology and environmental communication were used to establish the current version of the ENED-GEM (**Figure 1**), drawing inspiration from established frameworks such as the comprehensive GameFlow model (Sweetser and Wyeth, 2005) as well as various articles related to game-based learning. Additionally, central determinants for game-based enjoyment and motivation were identified as potential facilitators of learning through games. These were implemented into the model where applicable.

The ENED-GEM is a three-stage conceptual framework seeking to describe the psychological processes that occur before, during and after playing an educational game, with a special emphasis on factors that might influence pro-environmental behavior. ENED-GEM also takes into account the *external influential factors* that might affect a person’s willingness to engage in any of these three stages. The three stages of the ENED-GEM consist of a *motivational stage*, a *gameplay stage* and the subsequent *learning outcomes* from the gameplay stage. As a general rule it can be assumed that the model is largely linear, with the motivational stage coming before the gameplay stage and the subsequent learning outcomes that are gained from playing. However, it is important to note that a game could be played more than once, meaning that any new knowledge gained from playing the game the first time will likely be carried into the second stage of gameplay.

### External Influential Factors

Although the pedagogical properties of the game itself can be efficient in teaching on their own, it is important to also consider the interaction effects between the game and any psychological learning factors that exist outside of it. The ENED-GEM assumes that four external factors are influential in regards to motivating and steering gameplay. These factors are *pre-existing environmental tendencies, sociodemographic variables, player type* and *repeated play*.

#### Pre-existing Environmental Tendencies

Environmental awareness is known to stem from several sources, and subsequent environmental behavior or behavioral intention depends on a highly complicated framework of social, habitual, and personal factors (Kollmuss and Agyeman, 2002). People are motivated by both intrinsic (altruistic or moral) as well as extrinsic factors (rewards or incentives) to act in an environmentally friendly manner (De Young, 2000). Furthermore, to avoid the feeling of being regarded as incompetent or helpless in relation to a given topic, it is expected that individuals are motivated to learn, acquire information and actively participate in situations where they feel they should be involved or express interest, which is also applicable to the environmental domain (Kaplan, 2000). Taken together, the sum of an individual’s motivations to engage with environmental issues and topics constitute their *pre-existing environmental tendencies*.

Pre-existing environmental tendencies, such as attitudes toward specific environmental topics, are generally thought to affect a person’s behavior in regards to these topics (Fishbein and Ajzen, 1975, p. 335). It is therefore likely that the learning outcomes from playing an educational game about the environment are determined by the player’s pre-existing attitudes toward educational games and the environment in general, even before gameplay is initiated. A sufficient understanding of which personal factors are the most influential in determining environmental action does not exist, although factors such as knowledge of environmental issues and individual locus of control have been suggested as significant contributors (Hines et al., 1986/1987). However, based on existing research, the ENED-GEM assumes that an individual’s pre-existing environmental tendencies, such as their attitudes, beliefs and level of environmental knowledge, to a certain degree will influence their motivation to interact with an environmental game. They are also likely to play a part in the enjoyment and learning outcomes the players will gain from their gameplay sessions. As an example, it is likely that a person who is otherwise engaged and interested in bio-conservation perhaps would be more apt to play an environmental game with bio-conservation as its major theme than a person who is not involved in bio-conservation.

#### Sociodemographic Variables

Considering a player’s individual, social and cultural background can be important when examining for effects of educational games, regardless of the game’s theme or topic. In the case of gender for example, it is widely acknowledged that both men and women spend a great deal of their time playing games, and that certain gender differences tend to affect their motivations for playing. According to recent statistics based on more than 4000 American households, 67% of the households contained some form of gaming device (ESA, 2017). Furthermore, 59% of US gamers are male and 41% are female (ESA, 2016). Males generally tend to be more motivated by competition and achievements in their gameplay sessions (Williams et al., 2009), whereas females are shown to be more motivated to play games when no other leisure activities are available at the time (Chou and Tsai, 2007).

Different variations of game genres are also believed to induce flow states in players of a certain gender more easily than the other, such as in the case of how fighting and shooting games overall tend to appeal more to males than to females (Sherry, 2004). Females, on the other hand, show a tendency to be more attracted to the social interaction aspects of games (Hartmann and Klimmt, 2006), thus suggesting that they are more apt to play games with a multiplayer component rather than games solely meant for single players. The consideration of gender motivations in gameplay during the design phase of an educational game about the environment could positively affect the gameplay experience, and thus facilitate the learning outcomes generated by the gameplay sessions. Furthermore, gender is shown to have a significant role in regards to the level of positive or negative affect a person has toward the use of different types of educational games (Riemer and Schrader, 2015).

Another significant factor in gameplay is the age of the player. Although players are represented by all age groups, there are some differences between these that demand consideration. Firstly, the average player is normally assumed to be approximately 30 years old, and adults often play for longer hauls than younger players (Williams et al., 2008). Elderly players are also shown to enjoy games, especially when the game exists in a format other than digital and offers the possibility to socialize (IJsselsteijn et al., 2007). Intriguingly, elderly players have also been shown to exhibit a higher degree of self-reported well-being after playing digital games (Goldstein et al., 1997).

As for racial background, little consistent research exists to suggest any significant differences in the motivation to engage with games. In fact, designing games that are appealing across highly diverse audiences has been shown to be possible, such as teaching about artificial intelligence through role-playing games (Sintov et al., 2016). Furthermore, some research suggests that players do not exhibit any tendencies to play significantly more or less depending on what national background they have (Williams et al., 2008).

#### Player Type

The quality of an individual’s playing experience also depends on what type of player they are, and a person’s player type often reflects individual motivations for gameplay. One of the most cited taxonomies of player types divides players into four distinct categories: *explorers, achievers, socializers*, and *killers* (Bartle, 1996). *Explorers* enjoy discovering as much as they can about a virtual world, *Achievers* set in-game goals and try to reach them, *Socializers* wish to expand their in-game social networks, and *Killers* seek to disrupt and sabotage the gameplay for others. What is immediately apparent from these descriptions is that people have various motivations and needs for playing games, and that a failure to implement game elements that might satisfy these needs would result in a lower degree of game enjoyment for a wide variety of people. In educational games, the final consequence of low game enjoyment could be lack of attention toward the learning properties of the game as well as the inability for the players to enter a flow state or becoming immersed.

It is very rare that an individual fits exclusively within just one of these player types, and it is generally more common to exhibit traits from several player types at once. However, understanding this taxonomy as an overall categorization of existing player types is important in order to understand the intrinsic motivation an individual has toward playing a specific game, as environmental games designed to appeal to these player types would be perceived as enjoyable by a large part of the known gaming audience.

#### Repeated Play

Repeated play, or the desire to keep playing despite facing serious adversity or even beating the game in its entirety, also serves as an important component of digital educational games in that it re-initiates gameplay and contributes toward repeated exposure to the game’s educational content. The initiation of the repeated play of a game is determined by its *replay value* or *replayability* (Kelle et al., 2011), a measurement for a game’s potential for continued use after its initial completion (Wolf, 2012, p. 524). Modern sophisticated games, such as *Dark Souls 3* (From Software, 2016), often have multiple potential endings, achievements and quests that are only obtainable if the player chooses to play through the game at least twice or thrice. The game difficulty is often increased drastically on the second playthrough, and the game design tends to be slightly different from the first playthrough due to subtle or major changes to the game world. These new gameplay elements are intended to motivate the player to initiate repeated play, and to gain more enjoyment from their gameplay experience.

Repetition in educational games also serves a potentially important function in memory retention and ultimately the specific use of this retained knowledge in a practical setting (Ruben, 1999). First and foremost, repeating a set of implemented strategies to overcome in-game challenges could and should eventually lead to some form of reward for the player (Coyne, 2003). The reward can come both in the form of breaking out of the game’s repetition loop by finding a strategy that beats the challenge and allows the game to proceed, or it could provide the player with a tool that makes them stronger or more capable of overcoming future challenges. The player eventually learns which strategies and tools to use, and retains these important insights for similar events in later gameplay. In the event that an educational game is meant to simulate or otherwise resemble a real-life setting, it should also be possible for the player to integrate and implement learned in-game strategies to overcome real-life challenges (Bogost, 2010, p. 236).

### Motivational Stage

The motivational stage of the ENED-GEM initiates as soon as a potential player becomes aware of an environmental game, and includes the sum of motivations (both intrinsic and extrinsic) he or she has toward playing it. Motivations to play environmental games stem from a variety of sources such as their pre-existing environmental and gaming knowledge, values, attitudes and beliefs about environmental issues, as well as their potential desire to replay a game to complete unfinished quests or unlock new endings (see “Repeated Play”). The externalized factor of *player type* also serves as a central determinant as to whether or not an individual feels motivated to play, in that different player types are motivated to engage in gameplay by widely different in-game elements.

#### Intrinsic Motivators

*Intrinsic motivation* refers to any activity that is inherently enjoyable, meaning that the act of performing the activity is a reward in and by itself (Ryan and Deci, 2000). Commercially successful games generally feature a wide variety of known intrinsically oriented player motivators that eventually factor into player enjoyment, and these factors normally account for the game’s eventual popularity on the market. McGonigal (2011, p. 49) writes that the four intrinsic rewards we as humans crave the most can be summarized as (1) *satisfying work*, (2) *the experience or hope of being successful*, (3) *social connections*, and (4) *meaningful activities to do*. To a person playing a satisfying and well-designed game, all of these factors can be fulfilled through the act of playing.

Playing games is often a satisfying voluntary activity in and of itself (McGonigal, 2011, p. 21). Social connections can be established through in-game chatrooms and forums dedicated to the game, and the experience of being successful arises from becoming stronger and overcoming increasingly difficult obstacles the game world contains. However, research on game design states that there are several other factors influencing the intrinsic motivation to play. These motivational factors include *player-focused* as well as *in-game* elements, and can be both *intrinsic* as well as *extrinsic*. Intrinsic *player-focused* motivators generally arise from the player’s own willingness to engage and interact with a game (McGonigal, 2011, p. 51), and include the ability for players to become immersed into the visual aspects or atmosphere of the game (e.g., Brown and Cairns, 2004; Ermi and Mäyrä, 2005; Jennett et al., 2008). Players also tend to be motivated to experience emotionally charged narrative transportation (e.g., Green et al., 2000) in which the players are gradually absorbed into the relatable aspects of the game’s storyline. Additionally, the ability to socialize through online interaction (e.g., Malone and Lepper, 1987; Yee, 2006) has been proven to be appealing to a great number of individuals and particularly to female players (Hartmann and Klimmt, 2006). Facilitation and enablement for these experiences to occur happens through the presence of a wide variety of intrinsic *in-game* motivators, which include high-quality aesthetics such as graphics and soundtracks (Schell, 2008, p. 42), an optimal level of challenge (e.g., Malone and Lepper, 1987; Garris et al., 2002) and smooth controls (Wang et al., 2009).

#### Extrinsic Motivators

In contrast to intrinsic motivation, *extrinsic motivation* refers to performing an activity that leads to some form of separable outcome or external reward (Ryan and Deci, 2000), thus meaning that the motivation to perform does not stem from the activity itself. Externally motivated gaming activities focused toward education about a specific topic are commonly centered around some form of externalized reward such as course credit rather than intrinsically motivating in-game factors such as those described above. A large body of literature suggests that extrinsic motivation has a strong negative effect on existing intrinsic motivation to complete interesting tasks (Deci et al., 1999), as well as potentially limiting creativity in individuals who feel they are being controlled by an outside source (Amabile, 1998). For an environmental game that is inherently interesting to the player, offering some form of reward to complete the game (e.g., course credits or monetary compensation) would thus be likely to ruin the player’s enjoyment of the game as a whole as well as possibly limiting the amount of autonomous and creative thinking necessary to solve the game’s challenges. This would be likely to happen in educational institutions such as schools, where educational games are played to gain external rewards such as extra course credit or as a requirement for passing a class.

In cases where the game-based learning outcomes themselves are considered extrinsic rewards, however, it is clear that both intrinsically and extrinsically motivating elements need to be considered as complementary rather than mutually exclusive (Garris et al., 2002). There is also a possibility that games that are initially introduced solely with a promise of externalized rewards could contain intrinsically motivating elements as well, thus leading to voluntary repeated play and enjoyment of the game.

### Gameplay stage

The gameplay stage of the ENED-GEM begins when the player has begun actively engaging with the game, and normally features a certain degree of emotional activation in the person playing. During gameplay, the player is *immersed* in the audiovisual and narrative aspects of the game, and a flow state is achieved in cases where the game is highly immersive. In cases where the game features a deeply intriguing narrative the player might also experience *narrative transportation*, where they are so deeply immersed in the story and the characters of the game that they establish an *emotional connection* with the game world.

#### Immersion

*Immersion*, otherwise known as *presence* (e.g., Weibel and Wissmath, 2011), is a commonly cited, yet poorly understood construct in game-based learning. A common description of immersion is the feeling of being so absorbed into an experience or task that the flow of time seems to go by faster than usual, bodily needs such as hunger or thirst are suppressed and physical surroundings seem to matter less than they did before (Brown and Cairns, 2004). Immersion shows a considerable overlap with the flow phenomenon (Section “Flow”), although immersion is more fleeting and less persistent in nature (Brown and Cairns, 2004). It is also common to separate between flow as the pleasurable involvement in an activity, whereas immersion refers more to the feeling of being part of a mediated environment (Weibel and Wissmath, 2011). Immersion can be divided into three distinct categories; *sensory, challenge-based*, and *imaginative*. *Sensory immersion* refers to how audiovisual stimuli directs the players attention to the game, *challenge-based immersion* occurs when there is a fair balance between the game’s challenge level and the player’s skills, and *imaginative immersion* happens when the player starts to somehow identify with the game characters (Ermi and Mäyrä, 2005). Immersion is furthermore theorized to evolve gradually from a stage where it is easily broken to more robust full immersion (Brown and Cairns, 2004). Immersion is often associated with the degree of knowledge acquisition taking place during gameplay (Garris et al., 2002), with some researchers theorizing that it leads to intense experiences which increase learning, interest and retention of information (e.g., Murphy, 2011). In research, Weibel and Wissmath (2011) concluded that immersion and flow are both positively affected by motivation, and in turn have a concrete effect on the enjoyment and performance of a given task or activity.

#### Flow

*Flow* is a concept used to describe the psychological phenomenon of being so immersed in an activity or action that nothing else seems to matter, and the subsequent enjoyment one gets from this experience (Csikszentmihalyi, 1990, p. 4). It is commonly thought to follow the immersive stage of gameplay (Weibel and Wissmath, 2011), as described in the previous section (Immersion) of this article. A person who is experiencing flow is said to be in a flow state, which is generally considered to be highly beneficial to a wide variety of learning outcomes (e.g., Shernoff and Csikszentmihalyi, 2009), as well as to intrinsic motivation (Ryan et al., 2006). The flow state is also well known and sought after in game design, where it is commonly shown to increase player enjoyment as well as steering the player’s attention to what is happening in the game (Schell, 2008, p. 118). Being in a flow state during gameplay is significantly linked to learning, such as through increased concentration, interest, and enjoyment of the learning activity taking place (Hamari et al., 2016).

One of the most common precursors to these flow states during gameplay is an even balance between the game’s difficulty and the player’s own skills (Johnson and Wiles, 2003), where the difficulty should remain slightly higher than the point of frustration to give the player a goal to aim for. On the contrary, bad usability and slow feedback have been shown to be detrimental to the flow state in gaming (Kiili, 2005). Bad usability could refer to a variety of issues arising during gameplay that would otherwise ruin the immersion of the gaming experience, such as a poor relationship between the game’s difficulty level and the player’s own skills, or glitchy game mechanics.

Another intriguing finding is that flow states experienced together with others tend to be perceived as more enjoyable than when one is in a solitary flow state (Walker, 2010), thus suggesting that including a multiplayer function in the game is likely to boost the in-game flow state experience in some cases. It has also been shown that allowing the game to feature a structure where the players can enter into teams and compete against one another can increase their learning frequency (Admiraal et al., 2011).

#### Narrative Transportation

A *narrative* can be described as a cohesive story featuring a beginning, a middle section, and an ending, which provides the reader with some form of information regarding the characters, scene, conflict, and resolution (Hinyard and Kreuter, 2007). When one gets involved in the narrative to the point where an emotional connection is established with the characters and other elements of the story, this is known as *narrative transportation* (Van Laer et al., 2013). Narrative transportation is not limited to written materials such as books or magazines, but may be applicable in other forms of media as well (Green and Brock, 2000). The ultimate goal of narrative transportation is to have a persuasive effect on the reader or listener (Van Laer et al., 2013), thus indicating its potential use in educational games. Narrative transportation is also shown to have a distinguished effect on a person’s real-world beliefs regardless of whether it is based on fictitious or scientific material (Green and Brock, 2000).

#### Emotions

A key motivation to partake in the use of entertainment media, such as games, is the desire to experience strong emotional activation (Bartsch and Viehoff, 2010). During gameplay, players often experience a wide range of powerful emotions ranging from fear and surprise to wonderment and personal triumph (Lazzaro, 2004). Emotional activation is known to play a significant role in learning and memorization. On a purely biological level, an individual’s emotional and memory systems, mainly the *amygdala* and the *hippocampus*, respectively, are closely interconnected, and memories formed during certain emotionally aroused states could therefore be more easily recalled from memory (Sylwester, 1994; Brosch et al., 2013). Additionally, our attention tends to prioritize information that could somehow be emotionally relevant to us (Brosch et al., 2013). Experiencing positive emotions is also known to broaden the scope of human attention (Fredrickson and Branigan, 2005), suggesting that the ability to focus on more informational material and possibly also comprehend it more fully is increased. Positive emotions such as amusement and excitement are furthermore cited among the most common emotional occurrences during gameplay (Bateman, 2008), meaning that games could foster learning by broadening attention through emotional activation.

While the role of emotions is important in certain aspects of learning, it is largely neglected in educational game-based research (Wilkinson, 2013). Game-based social and emotional learning has been shown to be highly motivating, especially for younger learners (Hromek, 2009). Additionally, games can create powerful scenes that allow the player to experience emotionally charged events in a simulated virtual environment. This could, potentially, prepare the players for a real-life equivalent of this situation. Some games such as *That Dragon, Cancer* (Numinous Games, 2016) which thematically introduces the player to a child’s fight against cancer, are designed especially to provide such emotional journeys for the player to experience and gain insight from.

#### The Importance of Social Interaction

The ability to initiate some form of social interaction (e.g., cooperation, socialization, and competition) in games has been shown to significantly predict game enjoyment across a wide range of disciplines (e.g., Malone and Lepper, 1987; Bartle, 1996; Jennett et al., 2008; Fu et al., 2009). Games that teach language skills, for example, are shown to be effective when the opportunity to socialize and interact through the game environment is encouraged (Berns et al., 2013, p. 29). Additionally, female players (Hartmann and Klimmt, 2006) and the elderly (IJsselsteijn et al., 2007) are more likely to find gameplay enjoyable if they are given the opportunity for social interaction. Based on these findings, environmentally oriented games would do well to integrate a social arena through which the players can interact with each other.

### Learning Outcomes

Once the gameplay stage is finished it is likely that a well-designed educational game, regardless of the subject it is designed to teach, should result in some form of learning outcome for the player. The nature of these learning outcomes will likely depend on what the game is designed to accomplish; some games merely increase a subject’s knowledge or awareness of a specific topic, while others provide tools and procedural instructions on how to solve certain problems or change the player’s behavior in a desired direction.

#### Semantic and Episodic Knowledge Gain

Games have been shown to affect an individual’s cognitive structure on a wide variety of levels, ranging from spatial cognition (Feng et al., 2007) to certain elements of visual processing (Green and Bavelier, 2007). One of the more intriguing aspects of educational games is their ability to impact the human declarative memory. Tulving (1972, 1985) divided the human declarative memory into two interconnected parts; the *semantic* and the *episodic* memory. Semantic memory revolves around the perception, use and understanding of words in a meaningful and coherent fashion, while episodic memory contains information about episodes or events a person has experienced. Alterations in the human memory happens after repeated exposure to various types of information, and information campaigns are decidedly one of the most common strategies in environmental communication as a consequence (Klöckner, 2015, p. 164). Furthermore, information is one of the key factors leading to environmental action (Hines et al., 1986/1987). Studies show that playing specific types of games can lead to such alterations in the hippocampal area and thus the episodic memory (Clemenson and Stark, 2015). Similar results are found in more semantically oriented games where the goal is to acquire knowledge about language, especially when central player enjoyment factors are identified by the participants themselves (Butler, 2015).

#### Increased Perceived Behavioral Control

Perceived behavioral control refers to the perceived ease or difficulty of performing some sort of behavior (Ajzen, 2002), and is shown to significantly predict the intention to engage in pro-environmental actions (Bamberg and Möser, 2007). A closely related psychological phenomenon, locus of control, refers to an individual attributing their ability to bring about change either by themselves (internal) or through factors such as governmental structure or significant others (external) (Hines et al., 1986/1987). Showing how to perform a desired behavior has a tendency to reduce the perceived difficulty of a task, as well as increasing the PBC over it (Klöckner, 2015, p. 165). Games have the distinct advantage over other forms of media in that not only are they capable of displaying the potential effects of behavior change visually, but they also allow the player to be in control of the situation through their in-game characters and personas. Assuming the role of a virtual character while immersed in an environmental game might provide the player with a new arena through which they can gain an understanding of how to overcome environmental barriers. Playing educational games can also, for some individuals with a high level of external locus of control, lead to an increase in internal locus of control and behavioral intention (Yang et al., 2016). In environmental psychology, the effectiveness of behavioral interventions greatly increases when they attempt to remove barriers for behavioral change (e.g., Steg and Vlek, 2009), which educational games are apt to do through visually displaying the tools the player needs in order to overcome such barriers. Providing tools that make pro-environmental behaviors easier is shown to have a lasting effect in other studies (e.g., Thøgersen, 2009).

Additionally, games often contain colorful characters that the player can identify with or digital avatars that the player can assume the role of (Klimmt et al., 2009). While they didn’t test for the role of self-efficacy, Fox and Bailenson (2009) found that participants in a virtual environment would work out more in real life if they observed their similarly designed digital avatars doing it first. A different experiment concluded that taking on the role of a superhero in a virtual reality game caused more prosocial behavior in the participants, likely due to how embodying superpowers in the game briefly shifted the participants’ self-concept into someone who is likely to exhibit these traits (Rosenberg et al., 2013). Some researchers also suggest that an individual tends to experience an in-game narrative more positively than didactic instructions about how to act in a given context. In health research, for example, a person is more likely to integrate their vivid and direct in-game character’s positive experiences toward a healthier lifestyle than when they merely receive basic instructions on how to become healthier (Lu et al., 2012).

## ENED-GEM Case Study – Fate of the World

In order to provide preliminary validation for the ENED-GEM, a case study of the environmental PC game *Fate of the World* (Roberts, 2011) was conducted. The game was chosen due to being a rather scientifically accurate example in its representation of the climate system, as well as featuring a high level of difficulty and focus on learning about the environment in general (Klöckner, 2015, p. 199). Before initializing the information gathering stage, an application asking for permission to use informant data from the Steam platform was sent to the NSD (Norwegian Center for Research Data) for approval. NSD approved the project, under the terms that the reviewers had to be contacted by the researchers if their reviews were to be cited individually. Steam does not allow communication between members who have not yet added each other to their lists of acquainted players, however, and as a result communication with the reviewers became impossible. The reviews were therefore analyzed collectively, so as to not identify individual reviewers. This form of collective analysis falls under NSD’s guidelines for approval.

The reviews from a popular gaming client (Steam, 2016a) were collectively analyzed in order to gain an understanding of which elements in Fate of the World did and did not provide game enjoyment and if the elements included in the ENED-GEM could be identified in how reviewers refer to one example of a complex environmental computer game. Additionally, one of the researchers played through two of the game’s scenarios in order to gain an understanding of the game’s mechanics and interface. This process took 2.5 h, and was conducted on a brand new stationary gaming computer in order to ensure that the game ran as smoothly as possible. It should also be noted that the version played by the researcher did not include the downloadable expansion known as *Fate of the World: Tipping Point*, which features a scalable difficulty curve in the form of an “Easy Mode” (Steam, 2016b).

### Fate of the World

Fate of the World (FotW) is an award-winning digital card-based global strategy game (Steam, 2016a). Released in 2011, it was created as a joint effort between independent game developer Red Redemption and Oxford University as an attempt to educate the public about the effects of global warming on humanity and the planet as a whole (Soothsayer Games, 2017). In the game, the player takes on the role of GEO (Global Environmental Organization) in order to implement worldwide policies and projects that are intended to prevent environmental disasters such as droughts, famines, and epidemics from happening. These policies are presented to the player in the form of cards, where each card has a different effect on the progression of the game. Every time a set of cards (policies) are chosen, the player must proceed to the next round in order for the cards to take effect. Going from one round to the next makes the game move forward in time (5 years each round), and the player normally wins if they have completed their in-game tasks before a specific deadline. Depending on how the player chooses to use these cards, the 12 nations of the world (China, Europe, India, Japan, Latin America, Middle East, North America, Northern Africa, Oceania, Russia, South Asia, and Southern Africa) will either praise or resent the GEO’s decisions. If a nation becomes too resentful of the policies in play, the GEO will lose control over that nation and can no longer interact with it. Losing too much support from the various nations will cause the player to lose the game. In order to win the game, the player has to complete a set of goals that are unique for each scenario or level of the game. In one scenario (The Rise of Africa), increasing the HDI (Human Development Index) of North and South Africa to 0.7 or greater is the only requirement necessary to win. In another scenario (3°) the player needs to reach a specific deadline (the year 2200) with global warming below 3°, while simultaneously keeping a close attention to the world’s HDI and avoiding the loss of landmark species. The game features nine scenarios in total, ending with the Dr. Apocalypse scenario where the goal is to *raise* the global temperature without losing control of the 12 nations^1^.

In addition to these nine scenarios, the Steam version of FotW features a set of 32 achievements (trophies obtained after completing specific tasks in the game) available to the player, ranging from simply completing each of the scenarios to globally banning coal and even causing global thermonuclear war (Steam, 2016a).

### Reviews

Up until June 13th 2016, the full set of available user reviews of FotW on the popular gaming platform Steam (*N* = 249) were analyzed in order to gain a general understanding of the game’s perceived pros and cons. The reviews are public, and can be accessed both through the Steam platform itself as well as through any form of Internet browser (Steam, 2016a). Out of the 249 available reviews, approximately 77% (*N* = 192) rated the game as an overall positive experience. Collectively, the 192 reviewers who rated the game positively had spent a total of 4604.7 h (*M* = 23,98) playing the game. By contrast, the 57 reviewers who rated the game negatively had spent a total of 577.1 h (*M* = 10,12) playing. Eleven reviews were written based on the beta version of the game as it went through development, and as such will be excluded from this analysis due to potential significant differences between the unfinished and finished versions. Other reviews were largely vague or generalized opinions about the game as a whole, featuring only short statements such as “good game” or “not fun,” and thusly did not contribute sufficient information to be included in the final analysis. Furthermore, there are no separate review forums for the original FotW and its expansion, Fate of the World: Tipping Point. It is therefore likely that some of the reviews are based on the original game, whereas others are not. Furthermore, any sociodemographic variables about the reviewers are unavailable, thus making it impossible to ascertain any differences in opinion based on these constructs.

### Procedure

First, a short text was published on FotW’s Steam forums to inform the reviewers about the research taking place, as well as to give them the opportunity to withdraw their review from the collective analysis (Motsaenggin, 2016). To prevent the risk of identifying users, the reviews were analyzed collectively rather than individually. This was done in compliance with guidelines from NSD (Norsk Samfunnsvitenskapelig Datatjeneste) regarding the ethical treatment of informants in social science research. Statements about FotW contained within the reviews were then entered into an Excel spreadsheet, and listed according to how frequently specific aspects of the game were mentioned across the user base.

### Thematic Categories

Due to the usage of public reviews in this study, the informants were not tasked with answering questions from the researchers. The platform where the reviews are submitted (Steam) does not allow direct communication between users who are not added to each others’ friends-lists. Consequentially, it would be impossible to conduct interviews with the informants in this setting. Statements that coincided frequently were arranged into thematic categories by one of the lead researchers in an Excel spreadsheet by hand, and analyzed in accordance with existing guidelines for thematic analysis provided by Braun and Clarke (2006). Data extracts from these statements were utilized as codes, and subsequently linked together to form themes. The most frequently recurring positive statements about the game were *challenge* (48), *thought-provoking content about the environment* (19), *realism* (10) and that the game appeared to be *generally well-designed* (6). More negatively oriented reviewers were more apt to describe the game as *unintuitive in terms of layout* (12), *too difficult* (11), *in need of a sandbox mode* (9) as well as being *boring to look at* (8). After sorting the individual arguments found in the reviews of FotW into an Excel spreadsheet and counting the number of recurring arguments, a total of three main thematic categories were found to be relevant for the ENED-GEM framework. Other thematic categories, while interesting, did not occur a sufficient number of times to be included in the final analysis. Other arguments were so closely related to the overall theme of other categories, and were therefore fused together with these in order to avoid loss of valid information. The following section is dedicated to highlighting each of the three identified main themes, and to relate these findings back to the theory presented in the first half of the article.

#### Theme 1: Challenging or Impossible?

Out of the 249 reviews that were analyzed, a total of 59 mentioned the game’s difficulty level. Forty-eight users praised the level of difficulty by generally wording it positively (e.g., “fairly challenging” or “difficult”), while 11 users considered the high level of difficulty to be more negative, using terms such as “frustrating” or “impossible to beat.” Several reviewers mentioned that their implemented in-game strategies seemed to fail constantly regardless of how they played their cards, and some users eventually felt depressed or bored with the game as a consequence. Some of the positively inclined reviewers were also openly stating that the game’s difficulty level might alienate some players who did not feel comfortable facing off against it, and that a large degree of strategic gameplay was required to overcome it.

According to flow theory, the level of optimal difficulty is important both in regards to game-based learning (Hamari et al., 2016) as well as perceiving the game as fun or immersive (Schell, 2008, p. 118). Failure to make the game optimally challenging for a large crowd of players could result in the game being put down and, as a consequence, for any learning outcomes to remain absent. Should the player be given the option of adjusting the level of difficulty according to his or her skill level in the game it is more likely that the player would remain in a flow state, and thus learn more from the gameplay session due to a more even dispersion of cognitive resources between enjoying the game and focusing attention toward the game’s educational properties. A high level of difficulty could also result in the player’s attention being directed toward other aspects of the game rather than the educational properties, such as implementing strategies to avoid losing the current scenario or the support of 1 of the 12 major nations. The high challenge level could also cause a lower degree of PBC in that the player generates an understanding of the world as “unsalvageable” or “doomed,” due to implementing strategies that fail to fulfill the requirements for winning the game’s different scenarios.

While the general challenge level of FotW’s planned sequel is set to be lower (Soothsayer Games, 2015), a high degree of challenge could also lead to repeated play. Repeated play is generally an indication that while the game is highly challenging, there are elements of immersion and motivation present that generate an interest in reattempting to beat the game rather than to give up. Additionally, repeated play allows for players to establish a complex connection between the game world and the real world (Bogost, 2010, p. 236). It is likely determined by highly subjective reasons, although a flow state has to occur before repeated play is initiated. As explained earlier, flow is the sphere of optimal difficulty where the individual has achieved a good balance between the difficulty of the task being performed and their current task skill level (Johnson and Wiles, 2003). In flow theory, the enjoyment one gets from performing a task is heavily presumed, but the difficulty of the task and the level of skill exhibited by the individual performing the task have received considerably greater attention in the literature.

Regarding the occurrence of voluntary repeated play, one can infer that the player perceives the game as fun in general, a demonstrably important element in commercially successful yet frustrating games such as the *Dark Souls* series. In this adventure game series the player faces punishingly difficult challenges from the very beginning of the game, and the challenge level rises steeply as the player progresses through the game world. Beating the Dark Souls games conventionally requires a deep and complex understanding of the game’s mechanics, and it encourages repeated play by letting the player experiment with how to overcome the game’s obstacles, such as by equipping different weapons and armor when facing enemies with certain strengths and weaknesses, or even summoning other players to help them out in battle. By introducing these enjoyable and motivating elements into the game, the player will likely be motivated to keep playing and to memorize recurring patterns that are featured within the game’s theme. In educational games about the environment it is likely that player enjoyment factors need to be considered as equally important to the game’s difficulty level and the player’s skills in overcoming these difficulties. To summarize, player enjoyment factors likely facilitate a player’s desire to keep playing and increase their skill level, even when facing serious adversity in the game itself.

#### Theme 2: No Sandbox – No Fun – No Learning!

A recurring complaint among the reviewers is the lack of an in-game *sandbox mode*. The term “sandbox” in gaming commonly refers to an open world where the player experiences a large degree of freedom in terms of exploring the virtual world present within the game (Bellotti et al., 2009). Reviewers who criticize this lack of personal freedom in the gaming landscape state that the existing interface of the game is boring or takes a long time to get used to, which in turn affected their gameplay experience negatively. This complaint was often made by reviewers who were more occupied by traditional game mechanics than those who gravitated more toward the scientific model the game was based on. The lack of a sandbox mode could, ultimately, terminate the entire gameplay stage of the ENED-GEM framework for individuals who feel that this particular gaming aspect is important, which in turn would be detrimental to any learning outcomes that would normally result from an enjoyable gameplay experience.

For an educational game, being appealing to the player is absolutely fundamental in order for the learning to take place, otherwise it risks being put down before any educational content comes into play (Sweetser and Wyeth, 2005). All too often, educational games tend to be perceived as being more dull than commercially successful games (DeNero and Klein, 2010), which could potentially undermine such important factors as flow and immersion during the gameplay stage of the ENED-GEM. A sandbox mode could, therefore, be an important component in creating an immersive game world in which the players can unfold themselves.

When referring to an immersive game world, a sandbox mode can be considered a conglomerate of various player enjoyment factors that are commonly present at the same time in the game setting. It is likely that a desire for a sandbox mode could therefore, by extension, signify a desire for the presence of more traditional gaming elements that positively reinforce game enjoyment. These missing elements constitute a significant part of the gaming experience that facilitates the intrinsic motivation to play, and in FotW’s case includes aspects such as narrative transportation due to the lack of relatable characters as well as the inability to interact with other players. Some of the more highly recognized game enjoyment factors in existing research have been mentioned earlier in this article, but several others are likely to exist. Those factors that are mentioned, however, are often missing in FotW, such as through the lack of interesting characters in the game’s narrative (Hinyard and Kreuter, 2007), an optimal level of difficulty (Malone and Lepper, 1987; Garris et al., 2002) and the option to be able to socialize with other players during gameplay (Malone and Lepper, 1987; Yee, 2006). An environmental game dedicating more resources and attention toward these gaming aspects should, according to research, lead to a higher degree of immersion into the game and higher learning outcomes as a direct consequence.

#### Theme 3: Educational Game or Depressing Propaganda?

A final central theme emerging from the data were the opposing perceptions of FotW as either a thorough and comprehensive educational game about environmental issues in general on one side, and as depressing propaganda on the other. The reviewers who praised the educational value of the game commonly referred directly to the scientific foundation the game was based upon, whereas the reviewers who wrote the game off as a tool for spreading propaganda generally did so without referring to the science behind the game at all.

Positive reviews of FotW generally reflected the reviewers’ perception of the game as challenging but fair, complex, sophisticated and well-designed. A selection of four individuals from the more positively inclined reviewers also stated that they found it entertaining how you could be sadistic in your gameplay (such as by starting genocides to reduce the world’s carbon emissions), while others described the game as potential fun for “science-obsessed people.” Positive reviews were also more likely to mention that the game was thought-provoking and capable of increasing awareness of environmental issues, while simultaneously giving a realistic depiction of the complexity of the subject matter. A large part of the positive reviews did, however, mention that FotW might be more suitable for individuals who are already interested in the subject matter before gameplay is initiated, and that other players might perceive it as a somewhat confusing and overly difficult strategy game. The more negatively oriented reviews generally reflect this statement.

Negative reviews of FotW describe the game as overly challenging, boring, depressing, and suffering from poor game mechanics. Also, from a total of six reviewers who found the game to be outright depressing, a few of them explicitly noted that they felt a sense of unavoidable doom as a consequence of ever-increasing environmental issues, and that no matter the strategy they implemented in the game to prevent said environmental issues from happening they seemed to lose the scenario regardless. It is possible to assume, based on the tone of these reviews, that the depressing reality portrayed in the game has led to some of the players being left with a reduced PBC in regards to certain pro-environmental actions. The players are commonly faced with environmental issues of varying intensity during gameplay, and are left with a sense that “nothing works” or “we are doomed anyway” when their strategies to counteract these issues fail. This is reminiscent of *learned helplessness*, a phenomenon in which individual efforts to circumvent an unpleasant situation decrease when the situation is perceived as uncontrollable (Abramson et al., 1978). To prevent such learned helplessness in educational games about the environment, it is important to avoid introducing too much information to the player at once. Instead, the game should focus on introducing the player gradually to the environmental issues that the world is facing. Failure to do so would likely result in information overload, which is detrimental especially to educational games played on the computer (Chen et al., 2011).

A lower degree of learned helplessness while playing educational games likely suggests a higher degree of PBC. PBC increases when the tools for circumventing a problem are provided (Klöckner, 2015, p. 165), a finding that FotW does not necessarily address. If the players had been given hints about how to counteract the environmental issues as they arise rather than having to read up on one of the game’s many menus to find pointers to a solution, it is likely they would have implemented strategic thinking and problem-solving to overcome the challenge directly. Theoretically speaking, this effect could also be reinforced when an environmental game addresses specific environmental issues rather than the full picture of environmental issues in general, as indicated by research (Sandbrook et al., 2015).

## Implications for the ENED-GEM

The initial thematic analysis detailed above provides promising dawning evidence for the relevance and applicability of the ENED-GEM model in educational game design and -research. In future environmental games, it could be important to consider the inclusion of more traditional motivational gameplay elements that draws a larger crowd of players into the gameplay stage, such as the inclusion of a sandbox mode with a narrative, quests, and characters. This might give the player a greater sense of autonomy and options to act in the game world, shaping it according to their own gameplay strategies.

Accounting for the individual components of the ENED-GEM, the reviews suggested that a large part of the players benefitted from immersion, flow and emotional activation during the gameplay stage. Positively inclined reviewers stated that they found the game to be an overall pleasant experience, particularly due to it being well-designed, fairly challenging and thought-provoking. It is difficult to say whether narrative transportation played a significant effect on the players, in particular due to FotW’s relative lack of focus on a concrete storyline and relatable characters. The game also does not feature any form of multiplayer mode, thus making any measurements on social interaction between players impossible. Additionally, due to the Steam platform’s account privacy guidelines, it is difficult to check for gender effects since players are not required to list their gender in their user profiles. Lastly the reviews contain no statements that directly suggest increased PBC, although a total of 19 reviewers state that the game *generally* “made them think” about environmental issues. 10 reviewers also stated that the game made them think explicitly about the *complexity* of environmental topics, thus suggesting on a very basic level that they benefitted from increased knowledge about the environment through their gameplay.

The game seemed to appeal particularly to the *achiever* and *killer* player types (Bartle, 1996). Achievers played FotW due to their love for the game’s high difficulty level, and were more apt to praise rather than criticize the demanding challenges that the game provided them with. They did, however, also place special emphasis on the fact that they understood how the difficulty could alienate other players, and commonly recommended FotW only to other players who were already accustomed to difficult games. In the case of the killer-type players, they generally seemed to recommend the game for purposes other than being environmentally friendly, such as the Dr. Apocalypse scenario. They also rated the game highly due to how certain game mechanics allowed them to be overly sadistic, such as by lowering the game world’s carbon emissions by killing off the majority of the population.

The ENED-GEM framework also supports existing research on intrinsic motivational elements in games, and their effect on player enjoyment. Reviewers who found FotW to be a positive gameplay experience tended to describe the game as more enjoyable, and in some cases even more educational than their more negatively minded counterparts. Positively inclined reviewers were also more likely to engage in repeated play than negatively oriented reviewers. One possible explanation for this is that more environmental-minded players are more capable of suspending their disbelief and outright accepting certain lacking game mechanics than less environmental-minded players in favor of a theme or subject matter they are occupied with from before. Less environmental-minded players are likely just looking for a more traditional gameplay experience where they can become immersed, unfold themselves in the game world, solve quests, implement their favored strategies to overcome challenges, interact with intriguing characters and perhaps also form a social network with other players. FotW’s general structure likely does not fulfill these needs for some players and, as a consequence, might alienate players who are less interested in environmental issues from before.

While the number of learning outcomes identified in the FotW reviews were limited, it is highly likely that other forms of learning could take place when playing environmental games. Some games, for instance, utilize the concept of roleplay and avatar customization, where the player is free to design and act out the role of a digital self that is separate from his or her real-life equivalent. A player’s avatar often has entirely different values, morals, attitudes, and beliefs than the player does, depending on how the avatar is designed and the whims of the player. However, such forms of roleplay have been shown to be effective in changing real-life attitudes in accordance with the role being enacted (Janis and King, 1954; Elms, 1966; Fox and Bailenson, 2009). Based on these findings, a player scoring low on pro-environmentalism might experience a consequential positive change in his or her real-life views about the environment when roleplaying a more environmentally friendly character. For research into environmental games featuring these factors, it is highly likely that such role play effects can be observable.

## Limitations to the Study

While the initial framework for the ENED-GEM looks promising for use in educational game design and -research, there are limitations in the study that need to be addressed. First, using reviews as informational sources can result in obtaining information only from individuals who felt very strongly, positively, or negatively, about the game. Focusing exclusively on Steam reviews will also result in the lack of knowledge about the sociodemographic variables of the informants due to how the review system is designed, and potential underlying differences in Steam users from other gamers in regards to skill level or personal background could also affect their opinions about the game.

A second limitation of the ENED-GEM framework is the variation in how much support each of the learning aspects get from the statements of the reviewers. While a change toward pro-environmental behavioral intentions is important for eventual behavior change, for example, this factor did not receive much support from the Steam reviews. While some reviewers stated that the game “made them think,” it is difficult to explicitly state that this suggests a change in behavioral intentions rather than, for example, an increased knowledge about environmental issues in general.

A third limitation of the ENED-GEM framework is the lack of research on the effects of gameplay on the *procedural memory*, which allows individuals to see the connections between stimuli and responses as well as to act adaptively to their environment (Tulving, 1985). Games that focus not only on the environmental issue itself, but also on the processes by which to solve it or attempt to make a difference, are perhaps more likely to be efficient in pushing individuals toward pro-environmental behavior than their knowledge-increasing counterparts. Failure to show the player the connection between the proposed problem and the tools with which to overcome that problem would likely result in a lower degree of PBC (Klöckner, 2015, p. 165). A future inclusion of procedural memory learning outcomes in the ENED-GEM framework might therefore be feasible.

Lastly, while it is not a direct limitation in and of itself, the version of the game played by one of the researchers might differ from the version of the game played by the reviewers. In addition to the official expansion pack known as *FotW: Tipping Point*, the game also has a series of fan-made *mods* where the game mechanics are customized to provide a better or more satisfying gameplay experience. A future researcher with technological experience might want to examine these modified versions of the game further in order to obtain a comprehension of how fan-made content could facilitate the gaming experience in educational environmental games.

## Future Research

Future research is needed in order to further expand upon the ENED-GEM, and insight from interdisciplinary fields is warranted. A closer examination of the impact of each individual factor in the model on behavioral change intentions is also required. Additionally, applying the ENED-GEM framework to future case studies of environmental games would provide a solid foundation for further validation of the model. Despite this, the initial thematic analysis and suggested ENED-GEM framework holds promising suggestions for future research. There are, however, examples of environmentally oriented educational factors that the ENED-GEM found little evidence for during the FotW case study, and these require further expanding upon as environmental games grow more sophisticated. Examples include the use of games to change a person’s behavioral intentions, the inclusion of crucial *environmental communication strategies* through the gameplay such as *nudging* or *prompting*, and perhaps even more abstract psychological processes such as designing a game that can showcase the effects of the player’s actions directly on their environment, and simultaneously make the player draw a connection from the game world to real-world application. *Eco*, a game currently under development by Strange Loop Games, features game mechanics where the player’s actions all carry some form of consequence for his or her surrounding nature. One example is water pollution, where leftover waste from the game’s mining system seeps into surrounding bodies of water and thus having a large negative impact on the game’s plant and animal life (Strangeloopgames.com, 2016).

This article used the award-winning game Fate of the World as a case study, but environmental games are growing more sophisticated by the day. Current projects that hold some promise for future research on the topic of environmental issues and opportunities for change include digital games currently in development such as *Eco* (Strange Loop Games) as well as board games like *CO_2_* and the *Oil Springs scenario* of *Settlers of Catan* (Klaus Teuber).^2^ The ENED-GEM framework might serve as a useful tool for future researchers wanting to investigate such upcoming projects, especially in regards to the psychological processes that remain active during gameplay and facilitate learning.

## Ethics Statement

Ethics committee for approval: NSD (Norsk Samfunnsvitenskapelig Datatjeneste). This study was carried out in accordance with the recommendations of NSD. NSD deemed the project as exempt from written consent from the participants of the research due to how individual informants were anonymized and the information provided (public reviews of a media product) were analyzed collectively. Informants are entirely anonymous in terms of name, gender, social and cultural background, geographical location and other affiliations.

## Author Contributions

KF: Initial idea behind the research topic, literature search, article writing, establishment of the conceptual model (ENED-GEM). Lead author. CK: Advisory function, initial review of article content, general approval of article topic, other supervisory responsibilities. Co-author.

## Conflict of Interest Statement

The authors declare that the research was conducted in the absence of any commercial or financial relationships that could be construed as a potential conflict of interest. The reviewer JH and handling Editor declared their shared affiliation, and the handling Editor states that the process nevertheless met the standards of a fair and objective review.

## Footnotes

1 The exact educational properties of the Dr. Apocalypse scenario is unknown to the authors, and is not made explicit on the developing team’s website. It is possible that it simply serves as a scenario that is designed to test the player’s accumulated skills through several scenarios of gameplay, rather than educate him or her about the environment. Another possibility is that it attempts to educate the player about global warming by having them do the opposite of lowering the global temperature, thus reaching out to other learner- and player types.

2 Giochix.it
